# Genomic and health characteristics of crossbred dairy cattle in central Uganda

**DOI:** 10.3389/fgene.2025.1567910

**Published:** 2025-04-24

**Authors:** Enrique Sanchez-Molano, Robert Mukiibi, Valentina Riggio, Joel Ogwang, Leonard Kawule, Katali Benda, Peter Beine, Barend M. de Clare Bronsvoort, James Prendergast, Andrea B. Doeschl-Wilson, Adrian Muwonge

**Affiliations:** ^1^ The Roslin Institute and R(D)SVS, University of Edinburgh, Easter Bush, Midlothian, United Kingdom; ^2^ National Animal Genetic Resources Centre and Data Bank, Entebbe, Uganda; ^3^ Vetline Services Ltd., Kampala, Uganda

**Keywords:** dairy cattle, East Africa, crossbreeding, admixture, genomics

## Abstract

**Introduction:**

In Africa, dairy cattle contribute significantly to the economy; however, a substantial proportion of these animals are low-yielding indigenous breeds. To increase dairy productivity, crossbreeding with exotic breeds such as European Holstein and Jersey is becoming increasingly common. Uncontrolled crossbreeding practices, however, pose a risk to the genetic integrity of local breeds, as highly productive but potentially maladapted animals may replace indigenous populations. This study aimed to characterise the genetic structure of crossbred dairy cattle in Uganda

**Methods:**

We used admixture analysis, while also assessing genomic diversity and inbreeding levels. Additionally, we evaluated the utility of farmer-generated phenotypic databases by integrating them with genomic data to explore the impact of exotic breed crossbreeding on disease frequency.

**Results and discussion:**

Findings from this study show a strong influence of exotic breeds (e.g., Holstein) in Ugandan crossbred cattle, leading to lower inbreeding and observed homozygosity than those observed for indigenous breeds. Exploratory analyses of available disease records provided evidence of a strong survivor bias, likely linked to higher mortality rates from diseases such as East Coast fever. These results show the importance of investigating the genetic composition of farm animals, in order to develop informed and sustainable breeding strategies in African dairy cattle systems.

## 1 Introduction

The dairy cattle sector plays a crucial role in the food security and agricultural economy of Uganda, providing livelihoods for a significant portion of the rural population (Tijjani and Yetişemiyen, 2015). With an annual milk production of 3.85 million tons (DDA, 2023), the production system is dominated by smallholders (Ndambi et al., 2008) and characterised by a mix of indigenous, exotic, and crossbred cattle. A recent study in South-western Uganda (Waiswa and Günlü, 2022) suggested a relatively high proportion of dairy farmers using crossbred animals, mainly from Ankole and East African shorthorn Zebu with high productive European breeds (Kabi et al., 2016). Furthermore, according to government reports (UBOS, 2024), about 41% of the dairy cattle in Central Uganda are exotic and/or crossbred animals. Amongst the indigenous cattle, the Ankole breed accounts for 56.4%, whereas Zebu or Nganda are less prevalent (UBOS, 2024). The highest proportion of exotic and crossbred animals is found in the South-western region (32.6% of the total exotic/cross), followed by the Central region (22.3%). These two regions account for about 50% of the national milk production. Similarly, the lowest proportions are found in the arid and semi-arid Northern and North-eastern regions, where the maintenance costs of exotic or crossbred cattle are usually unfeasible.

Indigenous breeds such as the Ankole or the Zebu are well-adapted to local environmental conditions and diseases (Bahbahani et al., 2017), making them a vital asset for smallholder farmers but, being generally multipurpose breeds under low selection pressures, they have a relatively low milk production of about 1–2 L/day (Ema et al., 2018). Conversely, exotic breeds such as Holstein and Jersey and their crossbreds present higher milk production capabilities of five or more L/day (Ema et al., 2018). Whilst they can significantly enhance the income of dairy farmers, they are not well adapted to the local challenging environmental conditions (Marshall et al., 2019), thus showing increased susceptibility to diseases and higher mortality rates.

Although crossbreeding is a potential strategy to improve production while maintaining resilience to local conditions, recent studies have shown that it has not led to a significant wide-spread increase in milk production in the tropics (Bayemi et al., 2005; Galukande et al., 2013). Farmers are usually not involved in the design of breeding programmes and not informed of the potential loss of adaptive traits under unmanaged crossbreeding. Thus, this often leads to animals that do not align well with local conditions, and to a mismatch between the used exotic breeds (often Holstein Friesian) and the requirements of the local production systems (King et al., 2006). In addition, many programmes suffer from intermittent funding and a lack of supportive policies (Traoré et al., 2017), with positive results only achieved in well-managed research stations rather than smallholder farms. As such, this lack of management and planning can exacerbate existing challenges within the dairy sector, sometimes including reduced productivity compared to expectations, as well as increasing costs associated with animal healthcare (Ahozonlin et al., 2019).

Given the rapid growth and importance of the dairy sector in Uganda, with an increase in production of 37% from 2020/21 to 2022/23 contributing to Uganda’s foreign earnings *via* exports as well as its economic development (DDA, 2023), it is important to start considering additional strategies to foster improvement. This should be particularly pursued in Central and South-west Uganda, where most of the milk is produced. To inform these strategies, a deep understanding of the genomic diversity of the current cattle populations is essential. The Uganda National Animal Breeding Strategy and Action Plan (NAGRC&DB 2022; unpublished document) has set up, among its priorities, the need to characterise the existing farmed animal genetic resources, highlighting also the strategic importance of developing alternative sustainable breeding programmes aided by genomic tools and the conservation of local breeds. By understanding the genetic architecture of crossbred and local cattle, informed decisions can be made to balance productivity with other valuable traits identified by farmers. Furthermore, as the dairy sector continues to evolve, it is crucial to ensure that breeding practices align with goals of sustainability and resilience, particularly in the face of climate change (Galukande et al., 2013). To support the development of targeted data-driven breeding programs and to maintain farmers informed and involved in the development of breeding goals, it is also necessary to invest in creating and maintaining farmer-led databases to identify traits of interest and record productivity and health metrics across populations. By focusing on crossbred populations and using such platforms, researchers and policymakers can understand the implications of current breeding practices on both productivity and genetic diversity. This balanced approach is critical for the future of the dairy sector, ensuring that farmers are fully informed and that the sector remains resilient and productive in the face of ongoing challenges.

The main objective of the present study is, in line with the strategic priorities established by the Ugandan government in 2022 (NAGRC&DB, 2022, unpublished document), to investigate the genetic background of the dairy farm cattle population in Uganda’s Central region. Although Uganda is home to a variety of indigenous cattle breeds and widespread crossbreeding practices, the detailed genetic composition of these populations has yet to be thoroughly studied. As such, this study aims to i) pilot the investigation of the genomic composition of Ugandan crossbred cattle in the Central region and ii) assess the potential use of information available through farmer-led data platforms for future research in cattle health and cattle health genetics. For this purpose, we have used metadata from the LUNDA platform (Vetline, 2021), a digital platform initially developed to follow up the gestation period and insemination of farm animals (cattle, goats, sheep, and pigs). This tool was designed to support small-scale livestock farmers by providing a suite of services through SMS and web applications. Among these services, this platform allows farmers to track the health, breeding, and gestation cycles of their animals, including reminders for vaccinations, breeding, and disease management.

## 2 Material and methods

### 2.1 Animal genotypes, metadata and quality control

As part of this study, tail hair samples were obtained from 192 animals during routine visits by local veterinarians. 162 crossbred animals were sampled from the Central region of the country, covering 133 villages in three districts (Kampala, Mukono and Wakiso), where 10% of Uganda’s human population resides. Additionally, 10 animals from three of the most common local breeds/populations in the country were sampled, including Ankole (Kiruhura district), Ugandan Zebu (Soroti district) and Nganda (Masaka district). To avoid high relatedness of sampled animals, only a maximum of two samples were taken per farm. For the 162 crossbred animals, additional metadata was also collected *via* the LUNDA platform (Vetline, 2021), capturing age at collection time (thus serving as a measure of animal lifespan), breed (as reported by farmer), location (district, sub-county, parish and village), morphological traits (coat colour, presence or absence of hump and horns), live weight, fertility (number of calves born and their approximate average weight at birth) and disease records (binary records indicating if specific diseases were identified during the lifetime of the animal). The disease records included East Coast fever (ECF), common worms, anaplasmosis, foot-and-mouth disease (FMD) and lumpy skin disease.

Upon hair sample collection, each sample was individually placed in a hair card and treated with 72-80°C oven heating for at least 30 min to inactivate nucleases. Genomic DNA from each sample was then extracted from the hair roots and subsequently genotyped with the Illumina Bovine High Density (HD) genotyping array (approx. 777,000 Single Nucleotide Polymorphisms (SNP) across the genome). In order to ascertain the genomic ancestry of the crossbred animals while covering a wider range of potential ancestries, additional genotypes pertaining to 433 animals from the most common African breeds (including both taurine and indicine) as well as other commonly used exotic breeds (e.g., Jersey and Holstein) were obtained from a previous study (Riggio et al., 2022), aligned to the Illumina Bovine HD array, and merged with our data (for a total of 625 individuals). The considered breeds from this dataset were: Ankole, Karamojong, East African Shorthorn Zebu, Nganda, Sahiwal, Serere, Holstein, Jersey, Nelore, Gyr, Ndama and Sheko.

It is important to note that although the Karamojong and Serere populations are also zebuine of Ugandan origin (large and small types of shorthorn zebu from Karamoja and Serere in the North-eastern and Eastern regions, respectively), we used the term ‘Ugandan Zebu’ specifically for the ten zebu samples collected in this study, primarily from the Soroti district in the Eastern region. These three populations were analysed separately from the broader East African Shorthorn Zebu group, which included small-type animals from Kenya (Bahbahani et al., 2017), to investigate potential differences arising from local, regional, and country-specific breeding practices. Additionally, it is essential to highlight the rationale for including certain populations in the analyses and discussion, despite their current limited presence in Uganda (e.g., Nelore and Gyr). Their inclusion is justified by the relation with ancestral zebu populations that migrated from South Asia to Africa (Hanotte et al., 2002), thus serving as reference to explain the existing population structure and variation in Uganda and across the African continent. Moreover, they could be also valuable for identifying the genomic ancestry of contemporary Ugandan populations, reflecting not just necessarily recent crossbreeding but also the conservation of ancestral genomic regions within specific populations through local breeding goals and practices.

Map positions for the merged data were reported according to the UMD3.1 assembly. Only those SNPs with known position and located on autosomes were kept. Quality control of the genotypes was performed in PLINK (Purcell et al., 2007) in consecutive steps, and included the removal of duplicated positions, a SNP call rate threshold of 90%, minor allele frequency threshold of 0.02 and a sample call rate threshold of 90%. Further quality control also included a cut-off threshold of 0.75 for the pair-wise genomic relatedness (Yang et al., 2011), thus removing very highly related samples that may potentially result from duplication or cross-contamination errors.

The final genotypic data after quality control comprised 523 samples genotyped with 709,693 SNPs (with 157 of these being Ugandan crossbred farm cows). The breed distribution of these samples can be seen in the Supplementary Table S1.

### 2.2 Population structure and admixture

A principal component analysis (PCA) of the genomic relationship matrix was performed with GEMMA (Zhou and Stephens, 2012) to investigate population structure and relatedness patterns among the analysed populations.

To infer the number of ancestral genomic clusters and identify the individual degree of admixture from them, an unsupervised admixture analysis was also performed using ADMIXTURE (Alexander et al., 2009), which models the probability of the observed genotypes using ancestry proportions from a given number of ancestral clusters and allele frequencies.

In order to estimate the most likely number of ancestral clusters (K), a 5-fold cross-validation was used to estimate errors for different values of K, ranging from 6 to 20. To avoid bias due to genotype missingness and linkage disequilibrium (LD), SNPs with at least one missing genotype were removed using VCFtools (Danecek et al., 2011), and LD pruning was performed in PLINK (Purcell et al., 2007) assuming an r^2^ threshold of 0.3, window sizes of 50 SNPs and shifting steps of 5 SNPs. This led to a final set of 107,298 variants for the admixture analysis.

### 2.3 Genomic diversity and marker-based (SNP) inbreeding

Genomic diversity and SNP-based inbreeding was calculated for all populations (given the small number of samples from Ugandan Zebu as well as their similarity with East African Shorthorn Zebu in the previous analyses, these two populations were merged). Nucleotide diversity ( π ; Equation 1) and Tajima’s D (Equation 2) were estimated in windows of 1 Mb using VCFtools (Danecek et al., 2011) as follows (Carlson et al., 2005):

$$\pi =\frac{n}{n-1}\sum_{k=1}^{S}2p_{k}q_{k}$$ (1)

$$D=\frac{\pi -\theta_{s}}{\sqrt{Var\left(\pi -\theta_{s}\right)}}$$ (2)

where *n* is the number of haploid sequences, $p_{k}$ and $q_{k}$ correspond to the allelic frequencies (major and minor, respectively) at the *k* ^*th*^ SNP, *S* is the number of segregating sites (SNPs) and $\theta_{s}$ is the Watterson estimator, computed as $S/\left[\sum_{i=1}^{n-1}\left(1/i\right)\right]$ (Watterson, 1975).

Observed homozygosity within each individual was estimated from the diagonal of the allelic relationship matrix estimated (Equation 3) with the method from Nejati-Javaremi et al. (1997) as follows:

$${OH}_{NEJ}=\frac{\sum_{k=1}^{S}\left(\sum_{i=1}^{2}\sum_{j=1}^{2}I_{{ij}_{k}}\right)/2}{S}-1$$ (3)

where $I_{{ij}_{k}}$ is the identity of the two alleles *i* and *j* for the individual at the *k* ^*th*^ SNP across all *S* variants (Villanueva et al., 2021).

SNP-based inbreeding was estimated using the excess of homozygosity ( $F_{L\&H}$ ; Equation 4) proposed by Li and Horvitz (1953) and the method 2 ( $F_{VR2}$ ; Equation 5) proposed by VanRaden (2008) as follows:

$$F_{L\&H}=\frac{{SOH}_{NEJ}-\sum_{k=1}^{S}\left(1-2p_{k_{0}}q_{k_{0}}\right)}{S-\sum_{k=1}^{S}\left(1-2p_{k_{0}}q_{k_{0}}\right)}$$ (4)

$$F_{VR2}=\frac{1}{S}\sum_{k=1}^{S}\begin{array}{c} \frac{{\left(x_{k}-2p_{k_{0}}\right)}^{2}}{\left(2p_{k_{0}}q_{k_{0}}\right)}-1 \end{array}$$ (5)

where $p_{k_{0}}$ and $q_{k_{0}}$ correspond to the allelic frequencies (major and minor, respectively) at the *k* ^*th*^ SNP across all *S* variants in the base population, and $x_{k}$ is the genotype for the *k* ^*th*^ SNP coded as 0, 1 or 2 and accounting for the number of major alleles in the genotype. In this study, and for both inbreeding measures, the allelic frequencies in the base populations were assumed to be either: i) equal to the ones in the current genotyped population (similar to what commercial software such as PLINK do) or ii) equal to 0.5. The latter assumption is expected to produce similar estimates for both methods (Villanueva et al., 2021).

### 2.4 Runs of homozygosity (ROH) and ROH-based inbreeding

Runs of homozygosity (ROH) were computed for all populations using PLINK (Purcell et al., 2007), with East African Shorthorn Zebu and Ugandan Zebu considered as one population. The minimum number of SNPs constituting a ROH (l) was estimated using the formula (Equation 6) of Lencz et al. (2007) adapted by Purfield et al. (2012):

l=logeaSNloge1−H¯ (6)

where *a* corresponds to the percentage of false positive ROH (set to 0.05), S is the number of SNPs per individual (709,693), *N* is the number of individuals (523) and $\bar{H}$ is the mean heterozygosity across all SNPs (estimated as 0.3111). This resulted in a minimum number of 60 SNPs per ROH.

Additional parameters used to compute the ROH were the maximum allowance for one heterozygous and two missing genotypes per window, a minimum ROH length of 1 Mb, a maximum gap between consecutive SNPs of 500 kb, a minimum density of 1 SNP per 50 kb, a scanning window size of 60 SNPs (equal to the minimum number of SNPs, as recommended by Meyermans et al. (2020)), and a scanning window threshold of 0.05. By default, only ROH containing at least 100 SNPs were considered.

Subsequently, ROH-based inbreeding ( $F_{ROH}$ ) for each animal was estimated as $L_{R}/L_{A}$ (McQuillan et al., 2008), where $L_{R}$ is the total length of the genome covered by ROH and $L_{A}$ is the total length of the genome covered by all SNPs, with the latter being calculated as 2509482.75 Kb.

### 2.5 Health data and association with proportion of exotic genomes

Metadata available for the Ugandan crossbred farm cows in this study were analysed together with the admixture results, to explore whether the proportion of exotic genomes had any effect on disease records. For this purpose, data pertaining to common infectious diseases (ECF, common worms, anaplasmosis, FMD and lumpy skin disease) were analysed by fitting binomial logit models. The following fixed effect model (Equation 7) was used based on the information available:

$$logit\left(y\right)\;∼\;\mu +Xb+e$$ (7)

where y corresponds to the presence/absence of a given disease during the lifetime of the animal, μ corresponds to the intercept, *b* was the vector of the fixed effects and X its corresponding incidence matrix and e was the vector of random residuals. The fixed effects were geographical district (three levels) and being or not vaccinated against the disease (two levels), with the individual proportion of exotic genomes (Holstein plus Jersey, expressed in %), age at last record in years and weight in kilograms, fit as covariates. Significance of the fixed effects and covariates was assessed through Wald’s test.

## 3 Results

### 3.1 Population structure and admixture

**FIGURE 1:**
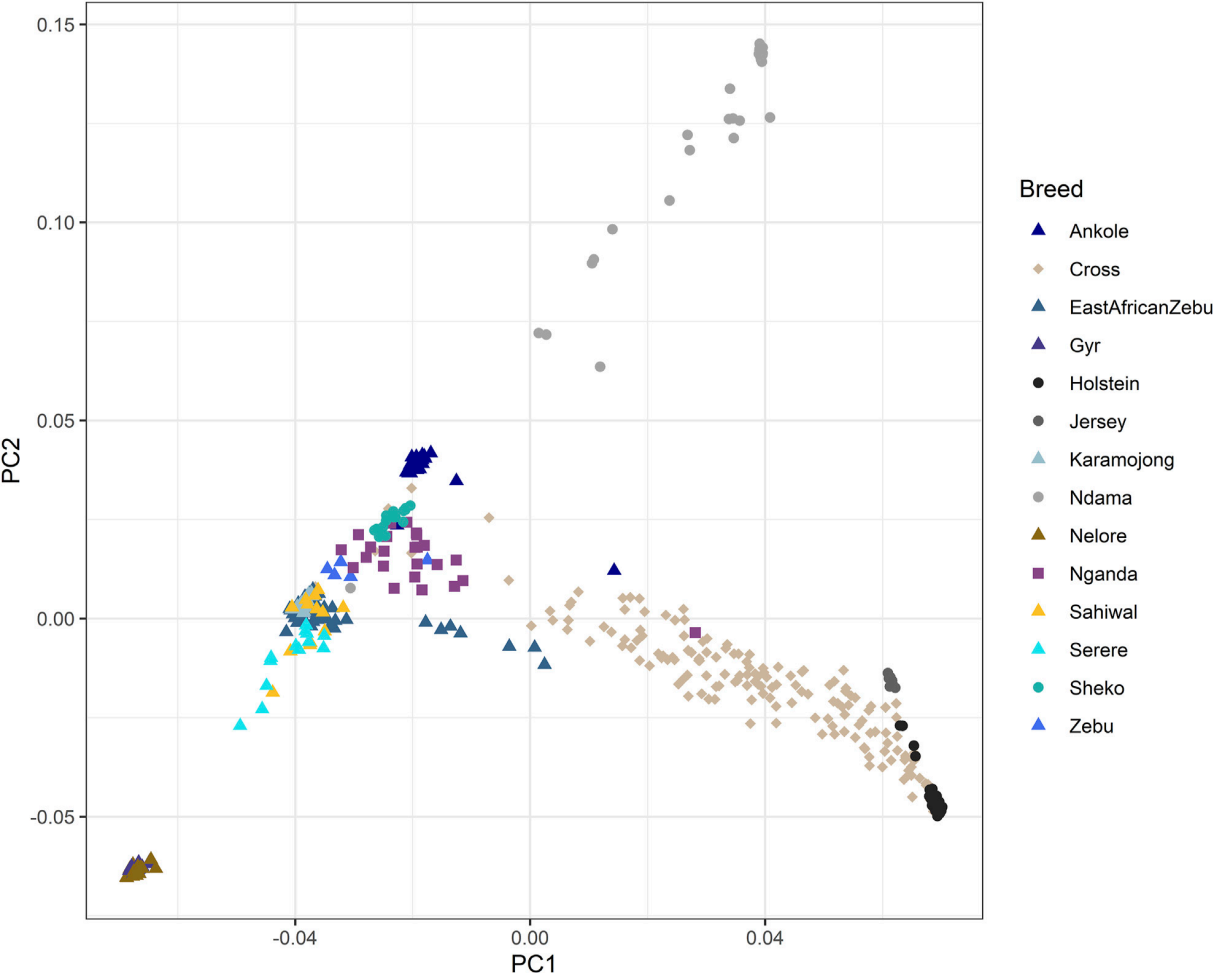
Principal component analysis of the genomic relationship matrix across all animals. “Zebu” corresponds to the Ugandan Zebu cows genotyped within this study, and “Cross” correspond to the crossbred farm cows genotyped as part of this study. Principal components 1 (PC1) and 2 (PC2) explained 17.7% and 3.9% of the total genetic variation, respectively.

The results of the PCA performed for the genomic relationship matrix across all animals are shown in Figure 1. Principal components 1 (PC1) and 2 (PC2) explained 17.7% and 3.9% of the total genetic variation, respectively. PC1 was mainly associated to the separation between European *Bos taurus* (Holstein and Jersey) and the African populations (mostly *Bos indicus* or crosses, with the exception of Ndama), while PC2 was mostly associated to the separation between Indian and African populations, with the extremes associated to the Indian *B. indicus* group (Nelore and Gyr) and the African *B. taurus* (Ndama). As expected, the crossbred farm animals genotyped as part of this study sat in between the zebuine group (East African Shorthorn Zebu, Ugandan Zebu, Karamojong, Sahiwal, Serere, Sheko), the Ankole and the European breeds.

**FIGURE 2:**
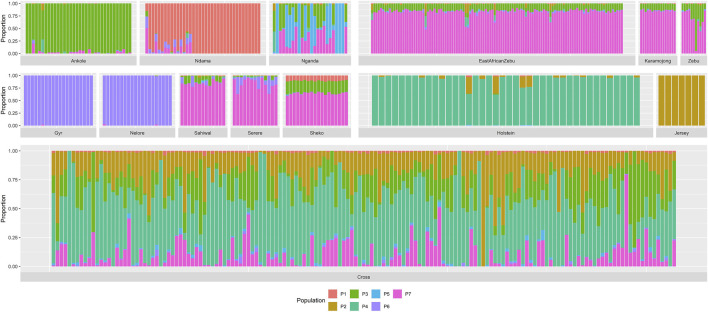
Admixture analysis for seven ancestral populations. “Zebu” corresponds to the Ugandan Zebu cows genotyped within this study, and “Cross” correspond to the crossbred farm cows genotyped as part of this study. Ancestral populations are mainly associated to Ndama (P1), Jersey (P2), Ankole (P3), Holstein (P4), Nganda (P5), Indian *Bos indicus* (P6) and the zebuine group (P7).

Cross-validation results from the admixture analyses indicated that the most likely number of ancestral clusters was 7 (cross-validation error of 0.51453). Although a slightly lower cross-validation error (0.51450) was estimated for eight ancestral clusters, this difference did not significantly impact our study, as it was due to some heterogeneity observed within the Ndama samples leading to two subpopulations within this breed (results not shown). Results from the admixture analysis with seven ancestral clusters are shown in Figure 2.

This analysis clearly identified ancestral clusters associated to the current populations of Ankole, Ndama, Holstein, Jersey, Nganda, the Indian *B. indicus* breeds (Nelore and Gyr) and the zebuine group (East African Shorthorn Zebu, Ugandan Zebu, Karamojong, Sahiwal, Serere, Sheko). Given the history of the cattle populations in Africa, some populations (such as Nganda and those within the zebuine group) presented varying degree of admixture with other ancestral clusters leading, in some cases, to similar patterns (such as the case of the zebuine group). Most of the crossbred farm animals presented relatively high proportions of exotic genomes, with an average of 48.6% Holstein and 19.9% Jersey, followed by a 18.5% Ankole and 10.1% zebuine. Among these animals, there were also some that were pure exotic, showing 99.9% proportion of either Holstein (2 animals) or Jersey (1 animal).

### 3.2 Genomic diversity and marker-based (SNP) inbreeding

**FIGURE 3:**
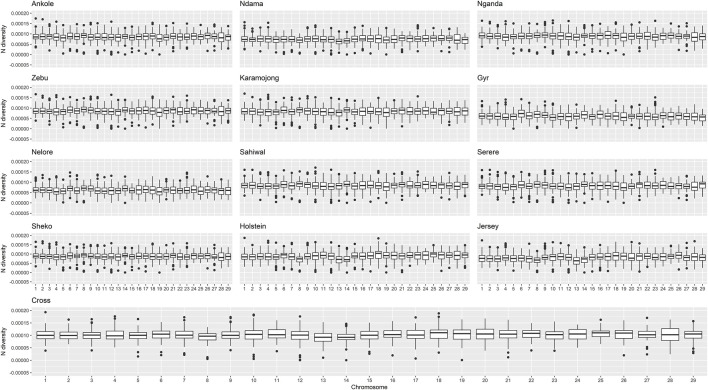
Distribution of nucleotide diversity per chromosome and population. “Zebu” corresponds to the Ugandan Zebu cows genotyped within this study, and “Cross” correspond to the crossbred farm cows genotyped as part of this study.

**FIGURE 4:**
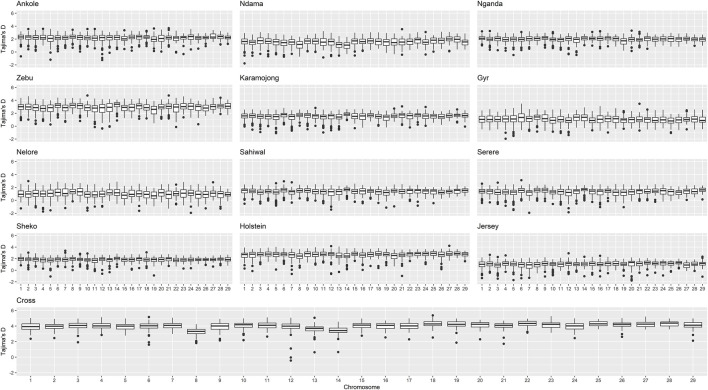
Distribution of Tajima’s D per chromosome and population. “Zebu” corresponds to the Ugandan Zebu cows genotyped within this study, and “Cross” correspond to the crossbred farm cows genotyped as part of this study.

The genomic diversity and Tajima’s D across the entire genome for all the studied populations are shown in Table 1, and were also plotted per region and chromosome (Figures 3, 4).

**TABLE 1:** Summary of genomic diversity ( π ) and Tajima’s D ( D ) per population. “Zebu” corresponds to both the Ugandan and the East African Shorthorn Zebu, and “Cross” correspond to the crossbred farm cows genotyped as part of this study. Standard errors of the mean ranged from 4.16E-7 to 5.1E-7 for genomic diversity and from 0.009 to 0.014 for Tajima’s D.

	π		D	
	Median (10^–5^)	Mean (10^–5^)	Median	Mean
Ankole	8.60	8.60	2.24	2.18
Ndama	7.39	7.41	1.57	1.53
Nganda	8.91	8.91	2.03	1.98
Zebu	8.63	8.63	2.99	2.89
Karamojong	8.38	8.39	1.62	1.53
Gyr	6.13	6.20	1.08	1.06
Nelore	6.19	6.25	1.04	1.02
Sahiwal	8.45	8.44	1.44	1.36
Serere	8.20	8.20	1.40	1.32
Sheko	8.74	8.76	1.88	1.84
Holstein	8.73	8.75	2.74	2.67
Jersey	7.94	7.99	1.11	1.03
Cross	10.02	10.01	4.03	3.97

Crossbred animals showed greater values than those observed for other populations. On average, Tajima’s D for Ankole, Zebu, Holstein and the crossbred animals showed values greater than two, being significantly different from 0 [based on a beta distribution at 95% confidence limit as in Tajima et al. (1989)] and indicating a lack of rare alleles, potentially associated with low effective population sizes. The Indian *B. indicus* group (Nelore and Gyr) showed smaller values of diversity and Tajima’s D compared to other populations. Within each population, chromosomes exhibited consistent patterns in both nucleotide diversity and Tajima’s D, with the exception of two outliers presenting negative values (close to 0) for Tajima’s D on chromosome 12 of the crossbred animals, potentially associated the heterogeneity of the crossbred population.

SNP-based inbreeding and observed homozygosity calculated for all the studied populations is shown in Table 2. As expected, when assuming the allelic frequencies in the base populations to be 0.5, both calculation methods for SNP-based inbreeding resulted in similar values. When assuming allelic frequencies in the base population equal to those in the current ones, the inbreeding coefficients were highly variable and difficult to compare. However, assuming allelic frequencies of 0.5 in the base population (maximum heterozygosity in the base population under Hardy-Weinberg), values were more comparable. The lowest inbreeding and homozygosity were observed for the crossbred farm animals, whereas most African breeds and populations showed values similar to Holstein. The Indian *B. indicus* group (Nelore and Gyr) showed the highest averages, followed by Jersey and Ndama. It is also interesting to observe that among the crossbred population there was an animal with a negative inbreeding coefficient (−0.216), associated to a very low observed homozygosity (0.392).

**TABLE 2:** SNP-based inbreeding coefficients and observed homozygosity per population. **“**Zebu” corresponds to both the Ugandan and the East African Shorthorn Zebu, and “Cross” correspond to the crossbred farm cows genotyped as part of this study.

	Base frequencies equal to current							
	Li and Horvitz				VanRaden			
	Mean	Variance	Min	Max	Mean	Variance	Min	Max
Ankole	-8.86E-03	2.12E-03	-2.51E-01	4.52E-02	-9.39E-03	3.97E-02	-1.29E-01	1.10E+00
Ndama	4.30E-02	2.91E-02	-3.85E-01	2.52E-01	4.30E-02	2.77E-01	-2.10E-01	2.65E+00
Nganda	-3.32E-02	2.74E-03	-1.52E-01	9.79E-02	-2.90E-02	6.71E-02	-2.57E-01	1.09E+00
Zebu	1.53E-02	5.34E-03	-3.13E-01	2.89E-01	1.45E-02	5.96E-02	-1.14E-01	1.32E+00
Karamojong	-2.70E-02	1.16E-04	-4.17E-02	-2.65E-03	-2.81E-02	2.80E-03	-1.24E-01	3.95E-02
Gyr	-1.02E-02	2.46E-03	-5.87E-02	1.61E-01	-1.17E-02	3.78E-03	-1.17E-01	1.08E-01
Nelore	-2.98E-02	1.66E-03	-8.63E-02	6.60E-02	-2.83E-02	5.21E-03	-1.23E-01	1.52E-01
Sahiwal	-4.53E-02	4.25E-04	-8.02E-02	-1.96E-02	-4.43E-02	5.14E-03	-2.04E-01	3.88E-02
Serere	-6.71E-02	4.84E-04	-1.07E-01	-3.32E-02	-6.40E-02	2.85E-03	-1.13E-01	4.36E-02
Sheko	-3.28E-02	8.77E-05	-4.72E-02	-1.66E-02	-3.19E-02	5.56E-03	-1.95E-01	6.37E-02
Holstein	-1.18E-02	1.38E-03	-7.71E-02	9.03E-02	-1.07E-02	1.94E-02	-1.61E-01	4.73E-01
Jersey	-5.05E-02	8.82E-04	-8.33E-02	-1.00E-02	-5.38E-02	2.03E-03	-9.69E-02	1.21E-02
Cross	1.49E-02	8.06E-03	-6.82E-01	2.40E-01	1.61E-02	1.39E-02	-3.55E-01	6.22E-01

	Base frequencies equal to 0.5							
	Li and Horvitz				VanRaden			
	Mean	Variance	Min	Max	Mean	Variance	Min	Max
Ankole	3.86E-01	7.88E-04	2.38E-01	4.19E-01	3.86E-01	7.88E-04	2.38E-01	4.19E-01
Ndama	4.99E-01	7.96E-03	2.75E-01	6.09E-01	4.99E-01	7.96E-03	2.75E-01	6.09E-01
Nganda	3.48E-01	1.11E-03	2.74E-01	4.31E-01	3.48E-01	1.11E-03	2.74E-01	4.31E-01
Zebu	3.94E-01	2.03E-03	1.91E-01	5.62E-01	3.94E-01	2.03E-03	1.91E-01	5.62E-01
Karamojong	4.01E-01	4.01E-05	3.92E-01	4.15E-01	4.01E-01	4.01E-05	3.92E-01	4.15E-01
Gyr	5.64E-01	4.57E-04	5.43E-01	6.38E-01	5.64E-01	4.57E-04	5.43E-01	6.38E-01
Nelore	5.51E-01	3.14E-04	5.27E-01	5.93E-01	5.51E-01	3.14E-04	5.27E-01	5.93E-01
Sahiwal	3.87E-01	1.47E-04	3.67E-01	4.02E-01	3.87E-01	1.47E-04	3.67E-01	4.02E-01
Serere	3.89E-01	1.81E-04	3.61E-01	4.09E-01	3.89E-01	1.81E-04	3.61E-01	4.09E-01
Sheko	3.72E-01	3.24E-05	3.63E-01	3.82E-01	3.72E-01	3.24E-05	3.63E-01	3.82E-01
Holstein	3.77E-01	5.25E-04	3.37E-01	4.40E-01	3.77E-01	5.25E-04	3.37E-01	4.40E-01
Jersey	4.45E-01	2.46E-04	4.28E-01	4.66E-01	4.45E-01	2.46E-04	4.28E-01	4.66E-01
Cross	2.87E-01	4.19E-03	-2.16E-01	4.50E-01	2.87E-01	4.19E-03	-2.16E-01	4.50E-01

	Observed homozygosity							
	Mean	Variance	Min	Max				
Ankole	6.93E-01	1.97E-04	6.19E-01	7.09E-01				
Ndama	7.50E-01	1.99E-03	6.38E-01	8.04E-01				
Nganda	6.74E-01	2.77E-04	6.37E-01	7.16E-01				
Zebu	6.97E-01	5.07E-04	5.96E-01	7.81E-01				
Karamojong	7.00E-01	1.00E-05	6.96E-01	7.08E-01				
Gyr	7.82E-01	1.14E-04	7.72E-01	8.19E-01				
Nelore	7.76E-01	7.85E-05	7.63E-01	7.97E-01				
Sahiwal	6.94E-01	3.66E-05	6.83E-01	7.01E-01				
Serere	6.95E-01	4.52E-05	6.81E-01	7.04E-01				
Sheko	6.86E-01	8.10E-06	6.82E-01	6.91E-01				
Holstein	6.88E-01	1.31E-04	6.68E-01	7.20E-01				
Jersey	7.22E-01	6.15E-05	7.14E-01	7.33E-01				
Cross	6.43E-01	1.05E-03	3.92E-01	7.25E-01				

### 3.3 Runs of homozygosity (ROH) and ROH-based inbreeding

**FIGURE 5:**
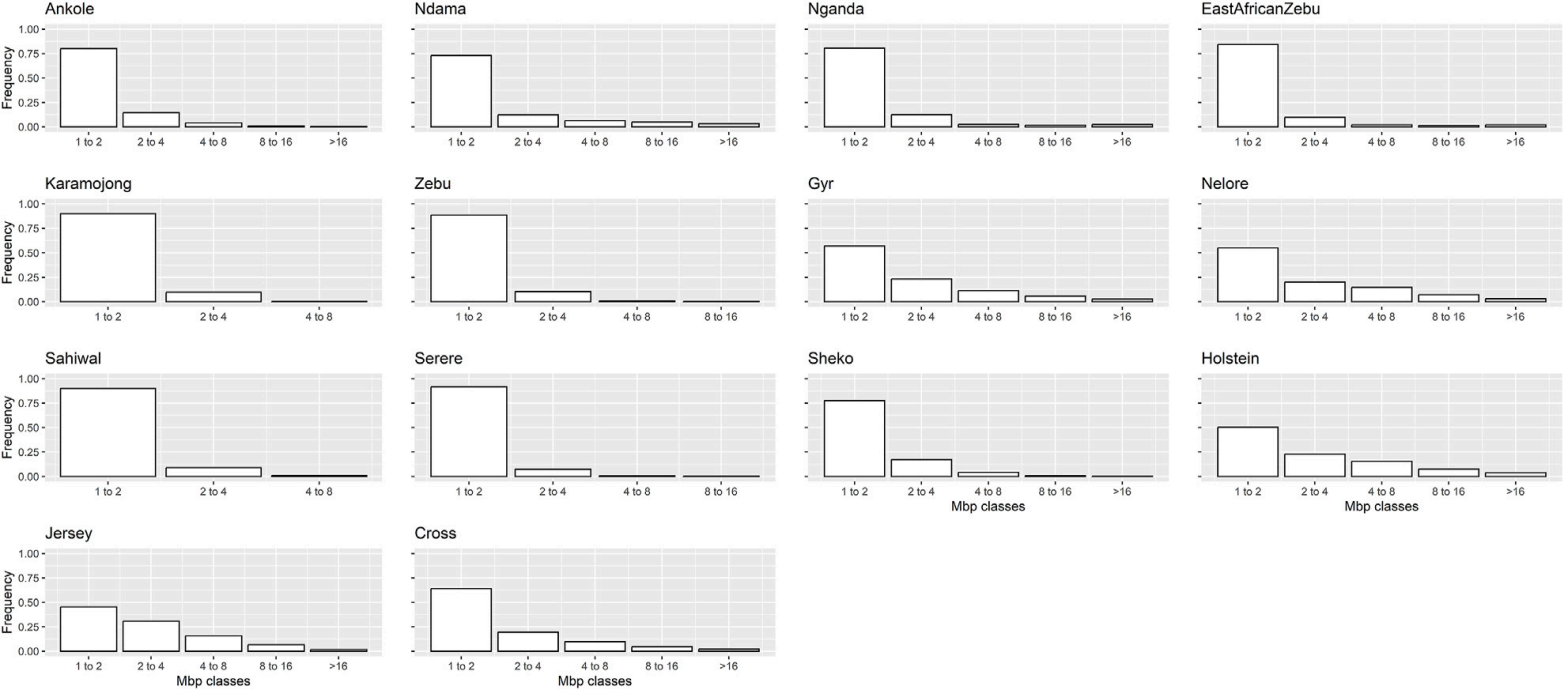
Frequency distribution of ROH per population, classified by size (Mb). “Zebu” corresponds to the Ugandan Zebu cows genotyped within this study, and “Cross” correspond to the crossbred farm cows genotyped as part of this study.

The ROH analyses detected a total of 20,062 ROHs across all populations and individuals. Table 3 presents. The average results for the ROHs per individual detected within each population are presented in Table 3, and the distributions of the detected ROHs per population based on their size, corresponding to five classes (1–2 Mb, >2–4 Mb, >4–8 Mb, >8–16 Mb and >16 Mb) is presented in Figure 5.

**TABLE 3:** Summary of ROH results per population. Summary statistics, including coefficient of variation (CV, in %) are presented for the total number of ROH detected per individual (N), the total length of the genome in kilobases covered by ROH in an individual (Tlength) and the average length of ROH per individual in kilobases (Alength). “Zebu” corresponds to the Ugandan Zebu, and “Cross” correspond to the crossbred farm cows genotyped as part of this study.

	N				
	Mean	Median	CV	Max	Min
Ankole	50.26	52.50	23.66	81.00	15.00
Cross	25.24	21.00	69.54	133.00	0.00
EastAfricanZebu	35.20	34.00	29.74	70.00	3.00
Gyr	53.20	51.50	24.15	91.00	39.00
Holstein	60.80	61.50	15.69	82.00	40.00
Jersey	121.86	121.00	11.31	143.00	105.00
Karamojong	35.25	33.00	16.30	46.00	26.00
Ndama	60.19	67.00	39.64	97.00	5.00
Nelore	47.75	46.50	19.25	69.00	34.00
Nganda	27.54	27.50	39.54	52.00	4.00
Sahiwal	29.23	31.00	31.39	40.00	5.00
Serere	22.00	24.00	43.91	34.00	2.00
Sheko	34.17	33.50	16.66	44.00	25.00
Zebu	34.18	33.00	27.12	53.00	15.00

	Tlength				
	Mean	Median	CV	Max	Min
Ankole	92552.66	90534.25	33.28	189460.00	23295.40
Cross	74429.91	43240.70	128.83	608898.00	0.00
EastAfricanZebu	78533.58	50145.90	149.45	745886.00	4972.29
Gyr	177612.82	132437.50	61.06	509791.00	65235.90
Holstein	247674.68	240364.00	31.88	453333.00	102213.00
Jersey	416398.00	429850.00	16.26	507760.00	325615.00
Karamojong	50377.78	47777.20	18.67	67901.00	34318.00
Ndama	181303.26	122718.00	91.87	655220.00	6575.37
Nelore	172716.34	145120.50	42.99	373899.00	87357.80
Nganda	66744.24	44729.30	146.47	460442.00	4919.63
Sahiwal	42171.72	44464.90	30.18	55513.10	5737.08
Serere	30905.58	33164.30	44.87	46477.30	2308.77
Sheko	62223.06	58543.45	29.90	113415.00	35205.00
Zebu	50714.13	45711.20	36.79	92870.80	19568.40

	Alength				
	Mean	Median	CV	Max	Min
Ankole	1820.14	1790.14	16.12	3157.66	1414.25
Cross	2458.30	2068.85	61.35	12178.00	0.00
EastAfricanZebu	1911.77	1458.74	91.25	11475.20	1179.15
Gyr	3142.96	2810.03	32.22	5602.10	1630.90
Holstein	4034.11	3713.96	26.66	7495.93	2330.05
Jersey	3407.37	3466.53	8.75	3877.13	3005.21
Karamojong	1425.32	1444.62	5.68	1544.15	1253.43
Ndama	2821.27	1806.02	74.43	9635.59	1315.07
Nelore	3535.68	3267.51	28.24	6559.63	2422.58
Nganda	2046.26	1500.30	90.34	8854.65	1229.91
Sahiwal	1436.14	1395.21	9.62	1679.17	1147.42
Serere	1396.87	1341.97	20.83	2323.86	1154.38
Sheko	1800.85	1783.82	18.13	2700.35	1408.20
Zebu	1453.67	1437.34	10.42	1752.28	1279.66

For the crossbred population, the average size of a ROH was 2,458 kb, with other African populations showing similar or lower average sizes. On the contrary, highly selected populations (such as Holstein, and Jersey) presented longer ROHs on average, with much higher proportions of the genome being also covered by ROH. In terms of individual ROH length variability, the coefficient of variation observed for crossbred cows was relatively high (61.35) and in line with those observed for Zebu, Ndama and Nganda, although the crossbred population presented the widest range among all populations, ranging from animals with no detected ROH to animals with ROH spanning more than 12 Mb.

In general, the distributions of the ROH classes based on size looked relatively similar, although in line with previous results, a higher frequency of longer ROH was found for highly selected populations (such as Holstein and Jersey).

ROH-based inbreeding estimates are presented in Table 4. Relatively low average inbreeding coefficients (≤0.02) were observed for several Uganda populations such as Serere, Ugandan Zebu and Karamojong. Slightly higher values, but still low, were observed for Nganda, Sheko and the crossbred Ugandan cows (0.02 ≤ F ≤ 0.03). The highest inbreeding levels were detected in the highly selected European *B. taurus* populations (Holstein and Jersey), followed by Ndama and the Indian *B. indicus* breeds (Gyr and Nelore). The highest coefficients of variation (≥100%) were detected for East African Shorthorn Zebu, Nganda and the crossbred farm animals in Uganda.

**TABLE 4:** Summary of ROH-based inbreeding results per population. Summary statistics, including coefficient of variation (CV, in %) are presented for the ROH-based inbreeding. “Zebu” corresponds to the Ugandan Zebu, and “Cross” correspond to the crossbred farm cows genotyped as part of this study.

	Mean	Median	CV	Max	Min
Ankole	0.037	0.036	33.282	0.075	0.009
Cross	0.030	0.017	128.829	0.243	0.000
EastAfricanZebu	0.031	0.020	149.445	0.297	0.002
Gyr	0.071	0.053	61.062	0.203	0.026
Holstein	0.099	0.096	31.885	0.181	0.041
Jersey	0.166	0.171	16.261	0.202	0.130
Karamojong	0.020	0.019	18.667	0.027	0.014
Ndama	0.072	0.049	91.871	0.261	0.003
Nelore	0.069	0.058	42.991	0.149	0.035
Nganda	0.027	0.018	146.474	0.183	0.002
Sahiwal	0.017	0.018	30.185	0.022	0.002
Serere	0.012	0.013	44.870	0.019	0.001
Sheko	0.025	0.023	29.899	0.045	0.014
Zebu	0.020	0.018	36.786	0.037	0.008

### 3.4 Health data and association with proportion of exotic genomes

Disease records were available for 144 crossbred animals out of the 157 that had passed quality control. While records on ECF were relatively balanced, with 63 cases (i.e., infected animals, accounting for 44% of the total), other diseases presented much more unbalanced data, with a number of cases ranging from five for FMD (i.e., 3.5% of the total) to 29 for common worms (i.e., 20% of the total), with anaplasmosis and lumpy skin diseases in between with 8 and 15 cases, respectively (pertaining to 5.5% and 15% of the total).

The distributions of admixture proportion of exotic genomes (Holstein and Jersey) present in each animal (fit as covariate), age in years and weight in kilograms are summarised in Table 5. Most observations were found within the districts of Mukono (51) and Wakiso (91), with only one observation in the district of Kampala.

**TABLE 5:** Distribution of proportion of exotic genome, age and weight in crossbred animals with phenotypic records. Proportion is given in the range 0–1, Age is measured in years and weight in kilograms.

	Mean	SE. mean	Median	St. dev	Max	Min	Skewness	Kurtosis
Prop. of exotic genome	0.690	0.019	0.700	0.226	0.999	2E-5	−0.757	0.551
Age	4.708	0.183	4.000	2.191	16.00	2.000	1.721	4.584
Weight	328.3	9.331	300.0	111.9	700.0	200.0	1.204	1.785

The percentage of exotic genetic introgression within an individual had a significant effect on ECF (P = 0.0106) and lumpy skin disease (P = 0.0486). The model coefficients for the covariable were −2.69 × 10^−2^ and −2.85 × 10^−2^, respectively, thus representing the amount of increase/decrease in the log-odds per 1% increase in exotic introgression. In both cases, the odds ratio was simply calculated by exponentiating these coefficients, resulting in values below 1 (0.973 and 0.972, with associated 90% confidence intervals of 0.954–0.994 and 0.945–0.999, respectively) and meaning that a 1% increase in the proportion of exotic introgression was associated to a 2.7% decrease in the odds of having ECF and a 2.8% in the odds of having lumpy skin disease. No significant effect was found for common worms (P = 0.243), anaplasmosis (P = 0.8904) and FMD (P = 0.0568).

To assess the statistical power of these analyses based on the sample size, *post hoc* power tests were conducted (Chow et al., 2017). These tests calculated the sample sizes required to achieve a specified type II error rate (i.e., the probability of failing to reject the null hypothesis when the alternative hypothesis is true), given the proportion of cases and controls observed for each disease and the estimated odd ratios. As there was more than one explanatory variable in the models, an inflation correction factor was used (Hsieh et al., 1998). The results from these tests indicated that a sample size of 144 records was sufficient to achieve a relatively low type II error rate (10%–15%) for the ECF analysis and a moderate error rate (30%–35%) for the Lumpy Skin Disease analysis. Assuming similar odd rations for other diseases such as FMD or anaplasmosis, results of the power tests showed that the larger imbalance between the number of cases and controls in the data resulted in much larger error rates, thus indicating a clear lack of power to detect significant associations for these diseases.

## 4 Discussion

The present study explored the genomic composition of crossbred cattle in Uganda, including comparisons with other African cattle breeds and exotic populations. We have also investigated whether the proportion of exotic genomes had any effect on health-related traits in Ugandan crossbred dairy cattle. Results from these analyses may serve to inform future breeding programmes aimed to enhance not only productivity, but also adaptation to local conditions.

The PCA analyses carried out in our study have shown a clear separation of European populations from non-European populations, mainly along the first principal component, which is expected due to the strong artificial selection carried out in the European breeds. The second principal component was mainly related to the separation of non-European populations, based on the taurine-indicine differences and the interbreeding between them. The admixture results, particularly those pertaining to the non-European populations, can be easily explained by the history and evolution of the cattle populations in Africa. The earliest African cattle is supposed to be the ancestral African *B. taurus*, arriving in different waves from the early domestication centres in the Middle East and expanding through the African continent (Hanotte et al., 2002). After this, the *B. taurus* population underwent two major introgression events by Asian zebu (*B. indicus*) from the Indian subcontinent across the East African coast and into the African continent (Mwai et al., 2015), with the latest event in the 19th Century being accelerated by the rinderpest epidemics that affected many *B. taurus* populations in East and South Africa. Thus, at present, the African cattle can be mainly divided into four major groups: *B. taurus*, *B. indicus*, Sanga (ancient *B. taurus* x *B. indicus* hybrids) and Zenga (Sanga x *B. indicus* backcross) (Mwai et al., 2015).

When focusing on the populations under study, the Nelore and Gyr are representative of the ancestral *B. indicus* groups that were originally maintained in Southwest Asia and contributed to the expansion of Asian zebu into East Africa (Zerabruk et al., 2012). In contrast, the Ndama population represents the ancestral African *B. taurus* lineage, predominantly found in West Africa and Congo (MacHugh et al., 1997). Other cattle populations occupy intermediate positions in the PCA, with admixture analyses strongly linking them to specific ancestral clusters. These include stabilised ancient *B. indicus* × *B. taurus* hybrids [e.g., Ankole (Taye et al., 2017)] and the descendants of the Asian *B. indicus*, such as the zebuine group encompassing East African Shorthorn zebu and regional populations like the Karamojong, Sahiwal, and Serere. Other populations with more recent origins show clear admixture patterns and are not fully classified as ancestral clusters in the admixture analysis. For instance, the Nganda breed represents a stabilised cross between Ankole longhorns and the small East African shorthorn zebu, placing it within the Zenga group (Masaba et al., 2024), while Sheko cattle exhibit admixture between Asian zebu and African taurine lineages (Bahbahani et al., 2018). Finally, the crossbred farm animals in Uganda revealed high proportions of exotic European genomes, mainly crossed with zebuine and Ankole. This is in line with the increasing popularity of crossbreeding with European genomes to increase productivity, which potentially could result in the replacement of indigenous breeds.

Nucleotide diversity, SNP-based inbreeding and homozygosity estimates for the populations under study were consistent with those reported for other cattle breeds (Gebrehiwot et al., 2021; Fabbri et al., 2024). As expected, the crossbred population presented the highest genetic diversity, coupled with the lowest levels of observed homozygosity and inbreeding. In contrast, the lower diversity and higher inbreeding observed in the other populations may be potentially attributed to their relatively small effective population sizes and/or strong selection pressures. Interestingly, the values observed for Holstein and Jersey, although consistent with previous studies (Melka and Schenkel, 2012), were similar to those seen in the African populations, despite the substantially stronger selection pressure typically applied in European breeding schemes. This similarity could be explained both by the inbreeding being accounted for in the European selection schemes and by most African populations, particularly the zebuine group, showing evidence of recent introgression and admixture. As such, it is not surprising that the lowest diversity and highest inbreeding was observed for the Gyr and Nelore populations.

When estimating inbreeding and genetic diversity, it is important to consider the assumptions and limitations of existing methods. Pedigree-based inbreeding estimates rely on the expected increase in homozygosity from mating between related individuals, ranging from 0 to 1. In contrast, SNP-based estimates are based on the observed homozygosity within individuals, but only a portion of this observed homozygosity is due to allelic inheritance from a common ancestor (identity-by-descent, IBD). To estimate IBD, the observed homozygosity has to be corrected by the allele frequencies in the base population (*p*_*0*_ and *q*_*0*_), typically assumed to be in Hardy-Weinberg equilibrium. There are, however, two main challenges in this correction: first, *p*_*0*_ and *q*_*0*_ are usually unknown, requiring assumptions about their values. Often, these frequencies are assumed to be either equal to those observed in the genotyped animals (ignoring genetic drift and selection), or equal to 0.5, representing minimum homozygosity under Hardy-Weinberg equilibrium (*p*_*0*_^*2*^ + *q*_*0*_^*2*^ = 0.5). Second, SNP-based inbreeding estimates measure the individual deviation in homozygosity relative to the one assumed in the base population, which can result in negative inbreeding coefficients for individuals that are less homozygous. In this study, inbreeding was estimated in a crossbred population of cattle that does not follow Hardy-Weinberg equilibrium and is highly outbred. Therefore, some individuals exhibited lower homozygosity than expected for the base population under Hardy-Weinberg, even when assuming minimum homozygosity.

Another method for estimating molecular inbreeding is the use of runs of homozygosity (ROH). Instead of estimating corrected IBD for individual markers, this relies on identifying segments of homozygous genotypes likely inherited from a common ancestor (IBD segments). Longer ROH are usually found in small, highly inbred populations or in those under strong selection (Browning and Browning, 2012), whereas shorter ROH are found in larger populations and may reflect old genetic bottlenecks (with recombination breaking the larger segments over time). The analysis of ROH provides more reliable estimates of whole-genome autozygosity, with ROH-based inbreeding calculated as the proportion of the genome covered by ROH and presenting a range limited between 0 and 1 (in line with pedigree-based inbreeding).

In this study, measures of ROH-based inbreeding were consistent with estimates in other African breeds (Terefe et al., 2023; Akinsola et al., 2024). When compared to other populations and likely linked to their low effective population sizes and/or intense selection pressures, the Holstein, Jersey, Gyr, and Nelore showed relatively high averages of ROH-based inbreeding, associated to higher frequencies of medium to long ROH segments (>2 Mb). Interestingly, the crossbred population also showed a relatively high frequency of these longer ROH segments but associated to a wider variation and lower average of ROH-based inbreeding. This finding may be explained by the relatively recent crossbreeding, where ROH segments inherited from both indigenous and exotic animals have not yet had time to undergo recombination, leaving the longer segments intact. To some extent, these results were mirrored by other highly admixed populations such as the East African Shorthorn Zebu, although specific regional zebuine populations (e.g., Karamojong, Sahiwal, Serere and Ugandan zebu) exhibited much less variation and lower inbreeding. These differences might be associated to the East African Shorthorn Zebu group corresponding to a mixture of animals from different subpopulations associated to differential breeding patterns and areas, as well as a few animals of this group presenting some introgression of European *B. taurus* (mainly Jersey).

The present study was also intended to serve as a pilot study to investigate the potential of emerging farmer-led health databases such as the LUNDA platform to serve as platform for genetic analyses of disease resistance or resilience of cattle when combined with genomic data. We investigated through simple approaches the phenotypic records for five common diseases identified in the database, together with the individual percentage of exotic genetic introgression estimated for the crossbred animals. For most diseases, the data was heavily unbalanced and, therefore, it is difficult to derive conclusions, as there was a lack of power to detect significant associations. However, for East Coast fever and Lumpy Skin disease, power analyses indicated that the available sample size was enough to achieve low to moderate type II error rates. It is recommended for future studies to increase the sample size to enhance statistical power and reduce the rate of false negatives, as well as aiming to achieve more balanced datasets in terms of the number of cases and controls.

In the case of the East Coast fever, data was relatively balanced and, interestingly, showed a significant association with the percentage of exotic introgression, where animals with lower percentage had a tendency to show the disease. While geography could be associated to potential bias in data distribution, with European animals being usually kept in high areas, where the incidence of ECF is lower, there were no substantial geographical differences in the areas where crossbred animals were sampled. Although the association between a lower proportion of exotic introgression in crossbred animals and a higher incidence of ECF might initially appear counterintuitive, it may be explained by the survivor bias (Anderson et al., 2011) and the high mortality and morbidity rates of the disease. Previous studies (Brocklesby et al., 1961; Ocaido et al., 2009; Gachohi et al., 2012; Wragg et al., 2022) have already indicated that the mortality rate for exotic breeds is around 90%, with death occurring usually within a week of the onset. On the contrary, indigenous cattle have a higher tolerance towards the disease, with high morbidity rates but much lower mortality (Kivaria et al., 2007; de Clare Bronsvoort et al., 2013; Mbole-Kariuki et al., 2014; Wragg et al., 2022). In this study, genotypes and health records were not collected simultaneously, as genotyped animals were alive at the time of sampling and had pre-existing disease records. Consequently, it is expected that affected animals with a higher percentage of exotic genomes were more likely to have died before they could be sampled. This may have introduced survivor bias, resulting in an overrepresentation of disease cases among animals with a lower percentage of exotic introgression in the dataset. This may have impacted to some extent the results from the admixture analyses. As such, it is recommended for future studies to not omit observations from deceased animals and collect, when possible, their genomic information. In addition, to mitigate survivor bias, farmer-led data platforms should prioritise collecting temporal disease occurrence records, capturing both diagnosed cases and those lost to follow-up over time. This longitudinal approach would provide a more comprehensive view of disease progression, enhancing the accuracy of survival estimates and risk factor analyses. Additionally, it would allow for a clearer distinction between true survival effects and biases introduced by incomplete case representation.

Finally, the current sample size of crossbred animals in this study is unfortunately too small to perform genomic association analyses, but a recent study (Wragg et al., 2022) found a genetic variant segregating in *B. indicus* associated to tolerance to East Coast fever. Given the potential for genetic variation linked to disease tolerance and the increasing prevalence of uncontrolled crossbreeding in low- and middle-income countries, which poses a risk to indigenous breeds, it is essential to understand how disease tolerance and resilience are associated with genomic variation in both indigenous and exotic breeds. To facilitate this, particularly in the absence of centralized management systems, it is crucial to foster participation in farmer-led databases such as the LUNDA platform. Aiming to enhance genetic improvement while maintaining genetic variation and conserving local resources, these databases will not only gather the necessary information for future government-led breeding programmes but will also integrate farmers into the decision-making process, identifying specific local needs and promoting sustainable production.

## 5 Conclusion

The present study on the crossbred cattle population in Uganda is one of the largest studies to investigate this type of populations in East African countries, serving also as a pilot study for the utility of farmer-led databases and showing the potential of genomic tools to inform breeding practices. Our admixture analyses have shown a large proportion of exotic introgression in the crossbred animals, identifying also distinctive genomic ancestries for other indigenous populations. While SNP-based inbreeding and homozygosity was lower for the crossbred population than for the indigenous populations, similarities in ROH distribution and ROH-based inbreeding suggest that the crossbreeding leading to these animals is both relatively frequent and recent. Finally, joint analyses with the phenotypic information available through the LUNDA platform showed a significant association between East Coast Fever incidence and the individual proportion of exotic genomes. However, the observed association is likely influenced by survivor bias, given the morbidity and mortality rates of the disease reported in previous studies.

Increasing participation in farmer-led databases, particularly in absence of government-led programmes, is critical both to facilitate further studies and to allow for the development of tailored breeding programmes. For example, the LUNDA platform contains information on traits outside the scope of this study, which would provide valuable information for genomic studies on production and fertility.

In addition, genetic monitoring over time and across regions is necessary to track genetic diversity, assess the long-term impact of breeding practices and support the resilience of Uganda’s dairy sector in a changing environment. Government and policymakers should be informed about the importance of genetic monitoring to maintain the diversity of cattle breeds and ensure sustainable development of the livestock sector.

## Data Availability

The datasets presented in this study can be found in online repositories. The names of the repository/repositories and accession number(s) can be found below: https://datashare.ed.ac.uk/, https://doi.org/10.7488/ds/7868.
